# Hidden multiple comparisons increase forensic error rates

**DOI:** 10.1073/pnas.2401326121

**Published:** 2024-06-10

**Authors:** Susan Vanderplas, Alicia Carriquiry, Heike Hofmann

**Affiliations:** ^a^Statistics Department, University of Nebraska, Lincoln, NE 68503; ^b^Department of Statistics, Iowa State University, Ames, IA 50011; ^c^Center for Statistics and Applications in Forensic Evidence, Ames, IA 50011

**Keywords:** forensics, error rates, database searches

## Abstract

When wires are cut, the tool produces striations on the cut surface; as in other forms of forensic analysis, these striation marks are used to connect the evidence to the source that created them. Here, we argue that the practice of comparing two wire cut surfaces introduces complexities not present in better-investigated forensic examination of toolmarks such as those observed on bullets, as wire comparisons inherently require multiple distinct comparisons, increasing the expected false discovery rate. We call attention to the multiple comparison problem in wire examination and relate it to other situations in forensics that involve multiple comparisons, such as database searches.

In forensic evaluations, a single conclusion often relies on many comparisons, either implicitly or explicitly. Multiple comparisons arise persistently when developing statistical methods to address scientific problems (1), and greatly increase the probability of false discoveries. Now that vast databases and efficient algorithms are routinely used in forensic evaluations to propose matches to crime scene items, the problem of close nonmatches (2) due to multiple comparisons becomes critically important. This often ignored issue increases the false discovery rate (FDR) and can contribute to the erosion of public trust in the justice system through conviction of innocent individuals. The multiple comparison problem is not new: it has been raised in the past with regard to DNA (3) and latent print evaluations (4). One of the root causes (5) leading to the wrongful accusation of Brandon Mayfield in the 2004 Madrid train bombing case was that the large size of the IAFIS database used to search for similar prints made it possible to locate “unusually” close nonmatches. As database size increases, so does the probability of finding a close nonmatch.

Compounding this issue, the use of algorithms also results in a large number of comparisons that are not obvious to the user. For example, the cross-correlation function (6), which computes the correlation for each alignment of two sequences, was one of the first measures proposed to quantify the similarity between two patterns in response to the 2009 NRC report (7), and continues to be used in many pattern searching algorithms to find the best alignment between two images and to quantify their overall similarity. Finding the best alignment often consists in sliding one surface across the whole length (for one-dimensional patterns, such as striations) or area (for two-dimensional sources, such as impression marks) of the other item while keeping track of the value of a similarity measure. This mirrors the forensic examination process: The examiner visually rotates and shifts items under a comparison microscope to align two surfaces. In order to avoid false accusations and the corresponding impact on public perception of forensics, we must address the problem of multiple comparisons in database and alignment searches and control their effect on FDRs.

Here, we consider the multiple comparisons problem that arises from a relatively simple toolmark examination: matching a cut wire to a wire-cutting tool. We describe the comparison approach, estimate the (minimal) number of comparisons that are needed to carry out the examination, and discuss how the FDR changes with the number of comparisons involved, using error rates derived from published black-box studies.

## Examination Process

A forensics examiner tasked with determining whether a wire in evidence was cut by a recovered tool will create one or more blade cuts, which are then compared to the cut surface of the wire recovered from the scene. These cuts are made in a sheet of material matching the wire composition and may be performed at multiple angles, as the angle of the tool to the substrate can affect which striations are recorded on the substrate surface. The blade cuts will then be compared to the wire under a comparison microscope, though eventually, automatic comparison algorithms may also be validated for lab use. Each side of each blade cut will be compared to each side of the wire; different tool designs have between two and four cutting surfaces in contact with the substrate.

## Methods: Calculating the Number of Comparisons

**Fig. 1.**
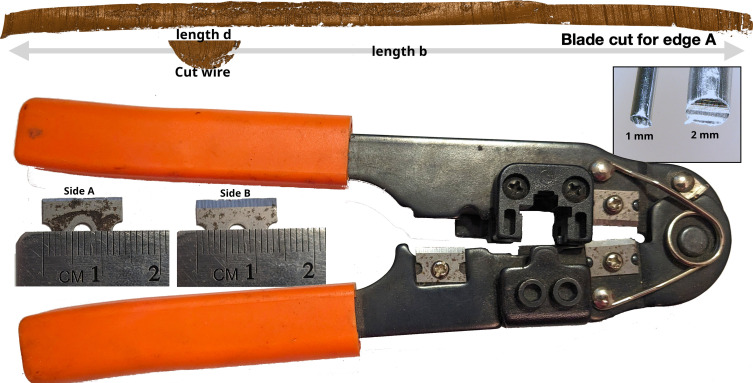
(*Top*) A comparison between a wire and a blade cut requires sliding the wire along the entire blade cut length to determine the best match (or whether there is a match). Surfaces shown are rendered 2D topographical scans of a wire and blade cut taken with a confocal light microscope. (*Bottom*) RJ45 Crimp tool with a 1.5 cm razor blade used for cutting. 1 mm and 2 mm diameter aluminum wires cut with the pliers are shown in a box in the *Top Right* corner.

In order to calculate the number of comparisons carried out in the course of one examination, we define b to be the length of the blade cut and d to be the diameter of the wire. We assume that the wire is covered with striations suitable for comparison across its full diameter d. If this is not the case, we reduce the value d. Both the blade and the wire are either digitally scanned at resolution r mm per pixel, or visually examined using a microscope with a digital resolution that can be expressed as r equivalent to the digital scan. An illustration of the sliding comparison process is shown in Fig. 1. Imagine that we move the cut wire along the blade cut in order to assess whether striations on the blade cut match the striations on the wire. We can move the wire unit-by-unit, or we can move the wire by its full length, with no overlap to the previous comparison.

The first option gives us the maximum number of comparisons (b/r−d/r+1), while the second option gives us the minimum number of comparisons b/d. In the first case, sequential comparisons share much of the same physical data and are highly related; in the second case, no data are shared between physical comparisons, and we can expect that they are statistically independent, though empirically there will be nonzero correlations due to physical similarities between striations. For simplicity, let us consider the number of comparisons to lie somewhere between these two estimates. Note that when b/d≈1, as in some toolmark comparisons, the upper number of comparisons goes to 1. Finally, we must consider the number of surfaces which must be compared: the wire may have one or two sets of striae and there may be two to four blade cut surfaces to examine, depending on the tool. This results in a multiplier of as much as 8.

### A Concrete Example.

Let us consider a wire-cutting tool with a 1.5 cm razor blade that meets a cast surface (one such tool is shown in Fig. 1); the wire is held against this rectangular cast surface as the blade is pushed into the wire, splitting it in two. This is a minimal scenario—the wire will acquire striations from one side of the blade, while the blade itself has two cutting edges, which we will call side A and side B. A blade cut of a sheet of aluminum will thus produce two striated edges corresponding to side A and side B which are compared to cut wires to assess similarity. We also have a 12 gauge aluminum wire (2 mm diameter) which may have been cut by the wire-cutting tool described above. Class characteristics, which are shared by all tools of similar manufacture, appear to match: There is a flat impression on one side of the wire corresponding to the cast metal backstop of the tool, and the wire is cut such that the blade and the backstop appear to be perpendicular (that is, the wire appears to have been cut with a tool of similar configuration). In this example, b=15 mm, d=2 mm, and there are at least b/d=7.5 comparisons between a wire cut and a blade cut. As there are two blade cuts (side A and side B), the minimal number of comparisons is 15, as these comparisons are nonoverlapping and independent (on average).

Assuming a resolution of 0.645μm per pixel, the maximum number of comparisons per blade cut is around 20,000; thus, we need 40,000 comparisons in order to find the optimal alignment between the wire and the blade cut. These comparisons are implicit in the calculation of cross-correlation, which is the first and often the only step used to quantitatively assess the similarity between striated evidence such as bullets, aperture shear, and firing pin impressions. Implicit comparisons are not unique to algorithms; an examiner would need to physically align the wire and the blade cut by searching along the length of the cut to visually match striations, performing the same process physically that the algorithm performs computationally. While these sequential comparisons are highly autocorrelated, and we cannot assume sequential independence when calculating the probability of an error, they serve as an upper bound on the number of comparisons which could be performed. As the number of comparisons increases, the probability of encountering a coincidental match increases. Statisticians call this the family-wise error rate E; it is an important quantity to control when conducting a series (“family”) of tests (8).

## Probability of False Discoveries

There are at least two components of the FDR: Identifying two pieces of evidence that have similar characteristics but are from different sources (a coincidental match) and procedural failures (e.g., lab process errors) (2, p. 50). In objective disciplines with standardized evaluation rules (e.g., DNA), these sources can be distinguished. However, in toolmark examination, no objective evaluation rules are used; examiners testify based on subjective rules for how much similarity is sufficient for an identification. Assuming that lab procedure errors are not a factor in studies, we use reported error rates from three open-set studies of striated evidence (9–11) to obtain a ballpark estimate of the coincidental match rate of a single wire-cut comparison. These studies have FDRs between 0.0045 (11) and 0.072 (10); pooling data from these studies weighted by sample size yields an FDR of 0.02. For a single-comparison FDR of e, the family-wise FDR for n comparisons, $E_{n}$ is $1-{\left[1-e\right]}^{n}$. Table 1 shows the impact the number of comparisons has on these published error rates. With an error rate of 0.007, as suggested by Bajic (9), examiners can make up to 14 comparisons, i.e., even the simple example in this paper exceeds an upper bound of 10% for the family-wise false discovery error. To conduct a search of a modestly sized database with 1,000 entries, the initial FDR cannot exceed 1 in 10,000 to guarantee a family-wise total false discovery error of at most 10%.

**Table 1.** Table showing the relationship between FDRs and the chance of a false discovery in N comparisons for a set of different FDRs and different number of comparisons

		False Discoveries (%) in N comparisons			
Study	FDR e	$E_{10}$	$E_{100}$	$E_{1,000}$	$E_{N}<0.1$
Mattijssen (10)	7.24%	52.8	99.9	100.0	1
Pooled error	2.00%	18.3	86.7	100.0	5
Bajic (9)	0.70%	6.8	50.7	99.9	14
Best (11)	0.45%	4.5	36.6	98.9	23
	1 in 1,000	1.0	9.5	63.2	105
	1 in 10,000	0.1	1.0	9.5	1,053
	1 in 100,000	10^−^4	0.1	1.0	10,535

The last column gives the number of comparisons allowed while ensuring a familywise false discovery percentage of at most 10%.

Under these constraints, the accuracy of an examination involving multiple comparisons between a wire and a tool will be low, as the number of candidate alignments that must be examined is high. Even the most innocuous example (small blade, only two cutting surfaces, and a relatively large wire) involves a minimum of 15 comparisons. Examiners would make cuts under multiple angles (12), increasing the number of comparisons and making a false discovery even more probable. As a result, it is questionable whether wire comparisons made under current protocols are reliable enough to be presented at trial.

Clearly, studies for wire evidence, and larger studies for striated evidence in general, are necessary. Moving away from binary assessments toward quantification of striation similarity and observed pattern frequency will also reduce the severity of this issue and allow examiners to assign unusual striation patterns more weight in the process.

## Discussion and Conclusions

Forensic practitioners often report the findings from their examinations in the form of a categorical conclusion reflecting a single decision. This is misleading when the decision relies on multiple comparisons which are not individually presented in reports or testimony. In this short contribution, we have shown that the implicit comparisons performed during forensic analysis of wire cuts increase the family-wise error rate.

We describe a simple scenario where a wire is cut using a two-sided blade, but findings apply to any situation where a forensic evaluation involves multiple comparisons, including, e.g., database searches. Forensic practitioners should understand how the number of comparisons can affect the accuracy of their final conclusion. We propose three strategies to enhance transparency and enable more reliable estimates of examination-specific errorrates.

First, examiners should report (or defense attorneys should request) the overall length or area of surfaces generated during the examination process, along with the total consecutive length or area of the recovered evidence. These pieces of information will take the place of b and d and facilitate calculation of examination-wide error rates.

Second, researchers should conduct studies relating the length/area of comparison surface to the error rate. For instance, we have pooled studies looking at bullet striations and firing pin shear marks because we could not find black-box error rate studies of wire cuts. The striated surfaces are of orders of magnitude different lengths but represent the best estimate of the error rate for striated materials. New studies should be designed to assess error rates (false discovery and false elimination) when examiners are making difficult comparisons.

Finally, when databases are used at any stage of the forensic evidence evaluation process (from suitability assessment and triage to reports which will be used at trial), the number of database items searched (or comparisons made) and the number of results returned must be reported. Additionally, the number of results used for further manual comparison should also be reported. For example, if a firearms examiner searches a local NIBIN database with 1,000 entries, requests the 20 closest matches to her evidence, and then carries out a physical examination of five exemplars from the list of 20, all of those values should be clearly reported to enable estimation of the familywise error rate. This will help make the multiple comparison issue accessible to everyone involved in evaluating the value of forensic evidence: examiners, lawyers, jurors, and judges.

## Data Availability

There are no data underlying this work.
